# 

**Published:** 1914-02-21

**Authors:** 


### CONTENTS.

| | PAGE |
|---|---|
| **A Review of Mr. Burns' Administration** ... | 549 |
| **The Cardiff Medical School** ... ... ... | 550 |
| **Hospital and Institutional News** ... ... ... | 551 |

T.R.H. Princess Christian and Princess Victoria of Schleswig-Holstein—St. George's Hospital—Understaffing at North Evington Infirmary—The Medical Officer's Position—Anoci-Association at Westminster Hospital—The Abuse of Nurses' Uniforms—Registration of Nurses—A Conversation that Produced £600—Hospital Treasurer Charged with Fraud—Sir Arthur Downes on Venereal Disease—Official Advice on Cheap Sanatoria—The Fatal Vogue of Eugenics—Wireless Telegraphy as a Hobby—School Meals and School Treatment—Radium and the Home Rule Bill—Infirmary Officers and Maternity Benefit—The Unfit Find a Vindicator—A Remarkable Retrospect in Aberdeen—An Ingenious Hospital Advertisement—Improvements at Colney Hatch Mental Hospital—Proposed New Nottingham Hospital—Dr. Julia Cock's Successor—A Question for School Medical Officers—X-Rays in Poor-Law Infirmaries—Proposed Register of Dispensers—This Week's Drug Market—To Our Readers.

| | PAGE |
|---|---|
| **Modern Methods Series.—III.** ... ... ... | 557 |

Cardiology—Abderhalden's Test—Leukæmia—Sciatica.

| | PAGE |
|---|---|
| **Pure Milk or Raw Milk?** ... ... ... ... | 558 |

A Sidelight on the Milk Problem.

| | PAGE |
|---|---|
| **Reports on Hospitals of the United Kingdom—Series III.** ... ... ... ... ... ... | 559 |

By Sir HENRY BURDETT, K.C.B., K.C.V.O.
The St. Albans and Mid-Herts Hospital.
Harrow Cottage Hospital.

[See over.]

### CONTENTS—(*continued*).

| | PAGE |
|---|---|
| **Buildings Contemplated** ... ... ... ... | 560 |
| **National Insurance Act Developments** ... ... | 561 |
| The Position of Employed Contributors Now Entering Insurance. | |
| **St. George's Hospital** ... ... ... ... | 563 |
| The Special Court of Governors. | |
| **The Central Poor-Law Conference** ... ... | 568 |
| Guardians and the Mental Deficiency Act. | |
| **Round the World** ... ... ... ... ... | 569 |
| Fellow-Workers and Their Doings. | |
| **Mental Hospital Magazines** ... ... ... | 570 |
| The Existing Product and a Gap to be Filled. | |
| **Institutional Needs** ... ... ... ... ... | 571 |
| **The Editor's Letter-Box** ... ... ... ... | 572 |
| The Open Basement—The Insurance Acts and the Hospitals—The Artificial Pneumothorax Operation—Staff Changes at Claybury. | |
| **The Profession of Medicine** ... ... ... | 574 |
| The Tragedy and Comedy of the Act. Post-Mortem Examinations. Further Light on Irish Problems. The National Medical Union. | |
| **The Institutional Worker** ... ... (Suppl.) | 1 |
| Designs for Coal Bunkers. | |
| **Our Bureau of Information** ... ... ,, | 2 |
| Questions for February. Questions Invited. The Sick and in Need. Employment and Training. The Housekeepers' Department. Miscellaneous. | |

